# Autism and Sexuality: Breaking Taboos and Embracing Desires

**DOI:** 10.1192/j.eurpsy.2025.780

**Published:** 2025-08-26

**Authors:** A. D. S. Moreira, R. Malta

**Affiliations:** 1 Serviço de Psiquiatria, Unidade Local de Saúde de São João, Porto, Portugal

## Abstract

**Introduction:**

Autism spectrum disorders (ASD) are a group of neurodevelopmental conditions characterized by impairments in social interaction, communication (both verbal and non-verbal), and repetitive behaviors. The global prevalence of ASD has increased significantly, with an estimated 28.3 million cases worldwide. Although many individuals with ASD have normal cognitive and language skills, difficulties with social interactions and understanding nonverbal cues can interfere with their ability to form romantic and sexual relationships, potentially leading to inappropriate behaviors and a distorted experience of sexuality.

**Objectives:**

This paper aims to review the literature on sexuality in individuals with ASD, focusing on typical sexual behaviors, sexual preferences, as well as hypersexuality and paraphilic fantasies and behaviors within this population.

**Methods:**

A non-systematic literature review was conducted, with article selection from PubMed using the keywords: “autism spectrum disorders”, “sexuality”, “hypersexuality” and “paraphilia”.

**Results:**

Studies have shown that adolescents with ASD experience higher rates of inappropriate sexual behaviors and gender dysphoria compared to neurotypical peers. Variants in sexual orientation, including homosexuality, asexuality, and bisexuality, are more prevalent in this population. Recent research indicates that sexual experiences, both alone and with others, are common among individuals with high-functioning ASD, with one study revealing that 47% expressed interest in having a romantic partner. Despite this, adults with ASD, especially men, are generally less likely to be in romantic relationships. They also exhibit more hypersexual and paraphilic fantasies and behaviors than neurotypical individuals. Hypersexual behaviors are predominantly observed among male ASD individuals, while paraphilias, such as voyeurism and fetishism, are frequently reported among both ASD men and women. Sadistic and masochistic fantasies and behaviors are also common in this group.

**Conclusions:**

The results indicate that individuals with ASD have a higher prevalence of sexual orientation variants and inappropriate behaviors compared to their peers. Although they have sexual interests and desires for relationships, their ability to express sexuality in a healthy way is hindered by communication deficits, social interaction challenges, and an unsupportive environment, exacerbated by inadequate sexual education. These challenges prevent many from fully embracing their sexuality. Specialized, evidence-based sexual education addressing the unique needs of individuals with ASD is essential for promoting healthier sexual behaviors and improving psychosocial well-being.

**Disclosure of Interest:**

None Declared

